# Round the Hospitals

**Published:** 1916-07-15

**Authors:** 


### THE MATRONS' AND SISTERS' DEPARTMENT.

### ROUND THE HOSPITALS.

THERE is no more difficult thing than successfully to co-ordinate and bring under one central government a number of small autocracies or republics. Government on a large scale, as in the case of empires, has necessarily to be simplified owing to its vast ramifications and responsibilities. These are best provided for and met by decentralisation. The building up of the College of Nursing, which to succeed will have to do with the whole profession of nursing in the United Kingdom, and ultimately in the Empire, is necessarily a complex, intricate, and slow process. We have from the first preached patience to everybody, and force is given to this teaching by the knowledge that at the moment the problems to be dealt with include not only an agreed Registration Bill, but the consideration of the exact relations to the College of outside bodies, more or less representative, the best of which will be well advised to throw in their lot heart and soul with the College and become a central portion of its machinery from the outset. We mention these obvious principles so that the nursing world may realise that everything of importance is proceeding steadily, and with commendable harmony and forbearance, so that the outlook for the successful conclusion of the initial stages and the unity of the nursing profession is sound and bright.

---

It takes many women to provide a matron. The first hospital matron in our experience, who reigned as a queen in a provincial hospital fifty years ago, was a woman of commanding presence and strong will. She was the widow of a merchant skipper, we fancy, and one reason which led to her appointment was the possession of a good supply of useful furniture, as the hospital she was to manage was domestically poor and ill-furnished. For thirteen years she ruled, and her departure was brought about with considerable difficulty owing to the terror she inspired in the members of the house committee of those days. She went in wrath, and selected as the day of her leaving the hospital 6 a.m. one Good Friday. She signalised her departure by two notable actions. First, she claimed to be the possessor of practically every piece of furniture the hospital contained that was worth anything whatever, and as there was no inventory or evidence of what was the hospital's she had her way and took it all. Secondly, she chose Good Friday morning, and, as the superintendent accidentally came to realise late on the evening before, instructed all her nurses and all the domestic servants and every woman worker that they too must leave the hospital with herself at 6 a.m. the same morning. This plan would have left 140 patients without attendants or staff to cook their food. By prompt action, however, this second exhibition of righteous indignation against the authorities who dismissed her ended in a fizzle. It is to her credit that in later years, after a further and somewhat chequered career as a matron of an even larger hospital, she saw her faults and failings and acknowledged them frankly and fully.

---

Hospitals have changed, and so have matrons, since those days. It is one of the remarkable and most exhilarating facts of the war, so far as nursing is concerned, that our women have proved equal to the position, and that the matron almost everywhere throughout our hospital system has risen to the emergency too, and proved in practice to be a most competent and indispensable officer. Matrons have had a most strenuous time, which has tested their abilities, their energies, their physical strength, and their spirit often to the breaking point. But in the result the matron has triumphed. Most of them have understood from the outset that our warriors, when needing hospital care, appreciate a homely hospital. We think that one of the noblest and truest definitions of the very best type of hospital matron was given recently in an outburst of grateful feeling by a soldier patient, who told his friends, "Our matron is a matron who endeavours to interpret her title by her actions." It is worth noting in this connection that military service means discipline, not autocracy, and the best discipline in war-time of every high official is wholly to sink self-consciousness in absorbed interest in the work for the sick.

---

Anno Domini proceeds slowly but surely as the world passes on its way. A life's work well done in circumstances that on the whole have been most congenial, and that may have produced many occasions for pleasurable contentment on the part of a worker when the age of retirement is reached, cannot be relinquished without wholesome regret, tempered by the honourable feeling that the hour has arrived. It is with mingled feelings of pride, recognition, and regret that we have to record the approaching retirement of Miss H. F. Hopkins, who for thirty years has been matron of the South Devon and East Cornwall Hospital. Hers has been an arduous, progressive, productive, developing, helpful spirit, and to her influence the Plymouth Hospital owes much more of its present satisfactory and high position than the world in which it works probably wots of. Thirty years is a long time to devote one's energies to one institution as a resident officer, and it is a remarkable fact, having regard to the manifold changes and developments which have taken place in the nursing and domestic departments of our voluntary hospitals, that Miss Hopkins should have continued to hold office with marked success for so long a period. When she came to the hospital it contained eighty-five beds, and at the present time, under war conditions, the number has been increased to 199. In 1886 there were only fourteen members of the nursing staff, with eleven private nurses for outdoor service. The nursing staff now numbers fifty-four, and we believe the private nursing staff has disappeared altogether.

---

It must be a gratification to Miss Hopkins to realise that nurses trained under her are now scattered over a large area of the nursing world, and those who have had the responsibility of finding and selecting workers for the war well know the South Devon Hospital nurses for their good training. That is saying much, especially when we remember that the probationers of to-day will no doubt have infinitely greater and better opportunities for becoming efficient nurses than those who preceded them in the years that are past. We are glad to know that the later working days of Miss Hopkins should have brought her the pleasure afforded by entertaining and making much of warrior patients. This will be a lasting and pleasant remembrance, and we should be grateful if those who govern the dispensation of honours, remembering the good training Miss Hopkins' nurses have exhibited in the war hospitals where they have been employed, will make it a point to see that her thirty years' excellent service shall be recognised by the R.R.C. decoration, which she has nobly earned.

[*Continued on p.* 375

**MATRONS' AND SISTERS' DEPARTMENT—(*continued*).**

A FORTNIGHT ago the Matron-in-Chief of Queen Alexandra's Imperial Military Nursing Service announced that nurses with three years' general training anxious to serve in military hospitals should at once apply to Miss Becher, R.R.C., War Office, Whitehall, S.W. This week we are asked by the Matron-in-Chief of the Joint War Committee to invite the attention of matrons and superintendents to the need that exists, owing to the advance of the British troops, for more trained nurses to serve under the Joint War Committee. It is essential that all such nurses should hold a three years' certificate, and they should apply to the Matron-in-Chief, Joint War Committee, 83 Pall Mall, London, S.W. Matrons and superintendents have already accomplished such wonderful work in rearranging their staffs so as to free their nurses for war claims that we make no doubt that, impossible though it may seem to do anything more in this direction, they will bend their whole energies to the task with a view to setting free every suitable nurse to take her part in nursing our brave warriors, either at home or abroad, as may be required. In this connection we direct attention to an advertisement in our columns this week asking for fully trained staff nurses for the extension of the 1st London General Hospital, Camberwell.

